# An Infrared Touch System for Automatic Behavior Monitoring

**DOI:** 10.1007/s12264-021-00661-4

**Published:** 2021-03-31

**Authors:** Qingqing Liu, Xing Yang, Ru Song, Junying Su, Moxuan Luo, Jinling Zhong, Liping Wang

**Affiliations:** 1 Shenzhen Key Laboratory of Neuropsychiatric Modulation and Collaborative Innovation Center for Brain Science, Guangdong Provincial Key Laboratory of Brain Connectome and Behavior, CAS Center for Excellence in Brain Science and Intelligence Technology, the Brain Cognition and Brain Disease Institute, Shenzhen Institutes of Advanced Technology, Chinese Academy of Sciences, Shenzhen, 518055 China; 2 University of the Chinese Academy of Sciences, Beijing, 100049 China; 3 University of Science and Technology of China, Hefei, 230026 China

**Keywords:** Automatic behavior detection, Elevated plus maze, Two-chamber, Looming, Foot-shock, Optogenetics, Fiber photometry, Heart rate and blood pressure

## Abstract

Key requirements of successful animal behavior research in the laboratory are robustness, objectivity, and high throughput, which apply to both the recording and analysis of behavior. Many automatic methods of monitoring animal behavior meet these requirements. However, they usually depend on high-performing hardware and sophisticated software, which may be expensive. Here, we describe an automatic infrared behavior-monitor (AIBM) system based on an infrared touchscreen frame. Using this, animal positions can be recorded and used for further behavioral analysis by any PC supporting touch events. This system detects animal behavior in real time and gives closed-loop feedback using relatively low computing resources and simple algorithms. The AIBM system automatically records and analyzes multiple types of animal behavior in a highly efficient, unbiased, and low-cost manner.

**Supplementary Information:**

The online version contains supplementary material available at 10.1007/s12264-021-00661-4.

## Introduction

When facing complex, volatile environments, animals behave in a species-appropriate manner for survival and propagation. Mammalian brains have evolved to orchestrate behaviors in response to a variety of stimuli. In neuroscience, behavioral feedback is an essential index of brain function. Neuroethological studies that focus on animal behaviors in a freely-moving state may reveal how and why the brain works [1]. In behavioral experiments with freely-moving animals, video recording is widely used. Typically, whole trials are recorded in which the position of the animal is tracked, and much analysis is done *post hoc* [1–3]. In many behavioral paradigms, such as the elevated plus maze (EPM) [4–7] and the two-chamber tests [8–10], the only information extracted from the video to describe animal behavior is its trajectory, or the position of the animal in each frame. However, analysis of video data requires a large amount of computational power, which puts demands on both hardware and the algorithms required to analyze animal behavior in real time. We have developed an automatic infrared behavior-monitor (AIBM) system as an alternative strategy to video recording for animal tracking.

In this system, a commercial infrared touchscreen frame is used to record real-time animal positions as ‘touch points’: the coordinates of the animal within the frame. Trajectory and speed are calculated in real time, and other behavioral parameters can be analyzed offline using MatLab scripts. Multiple cameras can be loaded in this system for further analysis of more subtle behaviors. Given the automation in data acquisition and manipulation, one experimenter can operate multiple systems simultaneously, enabling a high throughput of data collection. Moreover, potential effects due to experimenter bias are avoided, preserving a high degree of objectivity.

## Materials and Methods

### Animals

Male C57BL6/J mice (Beijing Vital River Laboratory Animal Technology, China) aged 8–14 weeks were group-housed (5 mice per cage) under a 12 h light/12 h dark cycle. All experiments were performed during the light cycle. Food and water were available *ad libitum* except for a fasted group, which were deprived of food for 24 h–26 h before a behavioral test. All animal experiments were conducted following protocols approved by the Institutional Animal Care and Use Committees at Shenzhen Institutes of Advanced Technology, CAS (SIAT-IACUC-200404-NS-LQQ-A1249).

### Hardware Design and Real-Time Data Manipulation

The AIBM device was designed using SolidWorks software (Dassault Systems, Waltham, MA). The behavior box consisted of an aluminum alloy profile frame, acrylic walls, and removable acoustic panels. An LCD screen positioned above was used to present visual stimuli in the looming stimulus paradigm and to provide lighting in other paradigms. The size of the behavior box was designed to fit the infrared touch frame. The infrared touch frame, the inner box, and the floor were separately placed on holders, which were fixed onto the aluminum alloy profile frame, such that they were easy to remove and replace. All behavioral apparatuses were designed as a replaceable inner box for the AIBM system.

The infrared touch frame (42 inches, 10 touch points, Guangzhou Yijing Electronic Technology Co., Ltd, Guangdong) was commercially available. It consisted of infrared LEDs and infrared receivers and was connected to a computer through a USB 2.0 port. Mouse positions were detected at 60 Hz *via* shadows falling on the receivers and calculated as the center of the enclosing rectangle parallel to the infrared touch frame. Real-time positions were recorded onto text (.txt) files using a C++ program, and further analyzed using MatLab scripts. Trajectory, speed, and acceleration were calculated in real time. When these parameters met pre-defined conditions, a signal was sent out through the serial port communication or the data acquisition (DAQ) card to trigger a stimulus or to other recording systems to timestamp an event for synchronization.

### Video Recording and Processing

For *post hoc* analysis, a digital video recorder (1920 × 1080, 60 fps, 360 Dash Cam, Beijing) was used. In experiments for accuracy evaluation, video frames were undistorted with Argus [11], and coordinates were matrixed with an inverse bilinear interpolation method using all 264 angular points of the grids in the analyzed region. Video data were manipulated in OpenCV. For real-time tracking, a PC camera (SY020HD-V1, S-YUE, Shenzhen) and two computers with different configurations (HEDY IABOX N20, HEDY, Guangzhou and Think Centre M910t-D565, Lenovo, Beijing) were used to record the trajectory of a remote-controlled car. The position of the car in each frame was calculated by a background subtraction method [12] using OpenCV. In the EPM and two-chamber tests, animal trajectories were extracted from video data using ANY-maze (version 4.96, Stoelting Co., Wood Dale, IL), and behavioral parameters were calculated using MatLab scripts.

### Position Detection and Spatial Resolution Test

Checkerboard pattern grids (4 × 4 cm^2^) were printed on advertising paper and pasted onto the floor of the AIBM system. A wooden cube (4 × 4 × 4 cm^3^) was used as a test object. The cube was put on each position and moved 1 mm, 2 mm, 5 mm, and 10 mm using a ruler for accurate positioning. These manipulations at each position were repeated five times and recorded simultaneously with both the infrared touch frame and the digital video recorder.

### Elevated Plus Maze Test

The EPM apparatus was made of frosted white acrylic board, with two open arms (23.5 × 5 × 17 cm^3^) and two closed arms of the same length extending from a central section (5 × 5 cm^2^) to form a right-angled plus. The distance between the ends of the open arms and the infrared frame was no less than 20 cm, and that between the sides of the open arms and the infrared frames was no less than 23 cm. The plus maze was elevated 85 cm above the floor. Individual mice were placed in the central section of the maze at the beginning of the experiment and their movements were recorded for 15 min. Mice in a group that underwent an acute stress procedure were individually restrained in centrifuge tubes (50 mL, with holes for air) for 120 min and then immediately transferred to the EPM apparatus. Mice in a control group were left in their home cages before the test.

### Two-Chamber Test

The two-chamber test apparatus was made of frosted white acrylic board and divided into two chambers (each 25 × 30 × 30 cm^3^) with an open doorway (5 cm wide and 30 cm high) between them. One of these chambers was baited with food. Regular mice chow (1 pellet) was placed on a clamp in one corner, whereas in the no-food chamber, one corner contained a clamp but no food. For analysis, we defined a food region (10 × 10 cm^2^) around the food corner and a similar no-food region in the other chamber. Food/no food chambers were counterbalanced across mice to reduce any potential bias inherent in the system. For each experiment, individual mice were placed at the door between the chambers, always facing the left chamber, and their movements were recorded for 20 min. Mice in the fasted group were deprived of food for 24 h–26 h prior to the test, and those in the control group were allowed *ad libitum* access to food. All mice were handled with cotton for 3–5 min before being placed in the apparatus.

### Looming Test

The looming test apparatus was made of acrylic and consisted of a refuge alley (40 × 10 × 30 cm^3^) connected to a circular open area (diameter, 50 cm; height, 30 cm). The floor was frosted white, the refuge walls were black, and the open area wall was transparent. A trigger area (diameter, 25 cm) was defined by a conceptual concentric ring in the center of the open area. Individual mice were placed in the center of the open area and their movements were recorded for no more than 15 min. Mice were given a 5-min habituation period to explore the apparatus and recover from any handling stress, during which time the trigger was not active. Following this, the looming stimuli trigger was automatically activated and triggered when specific mouse behavioral parameters fulfilled predefined conditions (movement within the trigger area at a speed < 0.15 m/s). The recording ended 2 min after looming stimuli were triggered. Looming stimuli were presented on an overhead LCD monitor (42 inches, refresh rate 60 Hz, AOC) displaying a gray background. The monitor was elevated 460 mm above the floor of the arena. Looming stimuli were generated using MatLab with Psychtoolbox-3. The looming stimulus was a dark disk that expanded from 2° to 40° in 300 ms and maintained this size for 50 ms before disappearing and was repeated 15 times at 30-ms intervals. In the negative control group, no visual stimulus was presented; that is, the trigger was not active.

### Foot-Shock Test

The floor used for the looming apparatus was replaced with a foot-shock pad for foot-shock tests. The test sessions, trigger area, and the predefined conditions were the same as in the looming test. Foot-shock currents of 0.5 mA were given to the shock group and no shocks were given to the negative control group. Foot shocks stopped as soon as a mouse entered the refuge and started again if it exited the refuge within 6 s after the initial trigger. Otherwise, foot shocks stopped 6 s after being triggered and the trigger was not active for the following 3 min.

### Stereotaxic Surgery

Mice aged 8 weeks–9 weeks were anesthetized with 1% pentobarbital sodium (i.p., 0.1 mL/10 g bodyweight) and fixed in a stereotaxic frame (RWD, China). Ophthalmic ointment was applied to each eye to prevent drying. The skull above the targeted areas was thinned using a dental drill and carefully removed. Injections were made using a 10 mL syringe connected to a 33-G needle (Neuros; Hamilton, Reno, USA), using a microsyringe pump (UMP3/Micro4, USA).

For optogenetic experiments, a total volume of 300 nL of either AAV2/9-CaMKIIa-ChR2-EGFP (BrainVTA, Wuhan) or AAV2/9-CaMKIIa-EGFP (BrainVTA, Wuhan) was injected into the superior colliculus at the coordinates −3.80 mm anteroposterior (AP), +0.80 mm mediolateral (ML), and −1.8 mm dorsoventral (DV). An optic fiber (200 µm, NA 0.22, Newdoon, Hangzhou) was implanted 3 weeks after virus injection. The coordinates for optic fiber implantation were −3.80 mm AP, +0.80 mm ML, and −1.5 mm DV. For fiber photometry, a total volume of 150 nL of AAV2/9-CaMKIIa-GCaMP6s (Taitool Bioscience, Shanghai) was injected into the dorsal periaqueductal gray at the coordinates −4.35 mm AP, 0 mm ML, +2.0 mm DV. An optic fiber (200 µm, NA 0.37, Newdoon) was implanted 3 weeks after the virus injection at the same coordinates. Mice were allowed to recover for 1 weeks–2 weeks before photometry recording.

### Optogenetics

Optogenetic tests were conducted in the looming apparatus. The trigger area and the predefined conditions were the same as those used in the looming test. Blue laser light (473 nm) was delivered with an Aurora–220–473 optogenetic system (Newdoon) through two optic fibers with a rotary joint (Newdoon) in the middle. Mice were allowed to explore the environment for a 5-min habituation period without triggering any laser stimuli. Following this, mice received 2.5 s laser stimuli (20 Hz, 5 ms pulse duration) when the predefined trigger conditions were met. Each mouse could trigger the laser stimuli no more than 5 times in 30 min. The interval between laser stimuli was no less than 3 min. The laser intensity at the tip of the optic fiber was approximately 15 mW.

### Fiber Photometry

Fiber photometry was performed alongside the looming test. The test sessions, trigger area, and predefined trigger conditions were as described above for the looming test. A dual-color fiber-photometry system (Newdoon) was used; it contained an excitation light (470 nm) and a reference light (410 nm). The intensity of the excitation light at the tip of the optic fiber (200 µm, NA 0.37, Newdoon) was approximately 60 µW. The intensity of the reference light was adjusted such that the signal was at the same intensity as the excitation light (GCaMP6s) signal. The GCaMP6s fluorescence was detected *via* photomultiplier tube (PMT). A LabView program was developed to control the laser intensity and the PMT recording. This program was used to record the fluorescence signal at 200 Hz and the behavioral event signal at 1 kHz. Before each fiber photometry recording session, the tip of the optic fiber was placed in a dark box and the background fluorescence signal was recorded for at least 1 min.

### Histology and Microscopy

Mice were euthanized with 1% pentobarbital sodium (i.p., 0.3 mL/10 g body weight) and then perfused transcardially with cold phosphate-buffered saline (PBS), followed by cold 4% paraformaldehyde (PFA, Sigma, Germany) diluted in PBS. Brains were removed and submerged in 4% PFA at 4°C overnight to post-fix, and then transferred to 30% sucrose to equilibrate. Brain sections were collected (40 µm) using a cryostat (CM1950, Lecia, Germany) and washed in PBS, stained with DAPI (1:5000, D1306, Thermo Fisher, MA), then coverslipped with aqueous mounting medium (Fluoromount™ F4680, Sigma, Germany). Sections were then photographed under a fluorescence microscope (Axio scope. A1, Zeiss, Germany) and analyzed using ImageJ.

### Blood Pressure and Heart Rate Recording

Blood pressure telemetry transmitters (PA-C10, DSI, MN) were implanted into mice following the surgical manual (391-0066-001, Rev. 60, DSI). Mice were allowed to recover for seven days after surgery before the blood pressure and heart rate were recorded during a looming test. Test sessions, trigger area, and predefined conditions were the same as those used in the optogenetics recordings. The receiver pads were grouped as one receiver and placed under the acrylic floor. Real-time pressure data were collected using Dataquest A.R.T. [TM] Silver Acquisition (Version 4.35, DSI). The event signals were delivered *via* serial port communication and recorded using the same software through a C++ virtual keyboard program.

### Data Analysis

Mouse positions were defined by the coordinates of the center of the mouse’s body within the frame. The percentage of time spent in a specific area was calculated from the ratio of the total time spent in that area to the total recording time. The numbers of entries into specific areas were counted when mice entered these specific areas over the whole recording period.

In the looming test, foot-shock, and optogenetics paradigms, distance to the refuge was calculated based on mouse trajectories. Latency to the refuge was the duration from the onset of looming to the time that the mouse’s body entered the refuge. Hiding time was calculated as the duration from the time that a mouse entered the refuge following looming to the time that it first left the refuge. The distance ratio was the length of the actual flight trajectory to the refuge divided by the straight-line distance from the trigger position to the center of the refuge-entry point. Instantaneous speed was calculated in real time every 16.7 ms during the entire recording session. Mean speed was the average of the real-time speed during the latency to refuge period, and maximum speed was the maximum speed during this period. The speed curve was the average speed curve from all mice in each group.

In fiber photometry recordings, the 1-min background fluorescence signal was fitted with a single exponential decay function after low-pass filtering of both the excitation and reference light channels, which was then used to deduce the background fluorescence of the fiber photometry recording. The Ca^2+^ fluorescence signal was calculated as the fluorescence recorded minus the background fluorescence. Background Ca^2+^ fluorescence was calculated from the single exponential decay fitting curve of the Ca^2+^ fluorescence signal after low-pass filtering. The Ca^2+^ response (ΔF/F) was calculated as Ca^2+^ fluorescence minus the background Ca^2+^ fluorescence, then divided by the background Ca^2+^ fluorescence.

For the measurement of physiological parameters, real-time pressure data were manipulated with Dataquest A.R.T. [TM] Silver Analysis (Version 4.35, DSI) and used to calculate blood pressure and heart rate, which were then analyzed using MatLab.

### Statistical Analysis

Data are presented using boxplots. All behavioral trials were treated independently for statistical analyses. Rank sum tests were calculated for comparisons between experimental and control groups. Statistical analyses were performed using MatLab. Asterisks indicate the level of statistical significance (*P <0.05, **P <0.01, ***P <0.001).

## Results

### The AIBM System

**Fig. 1:**
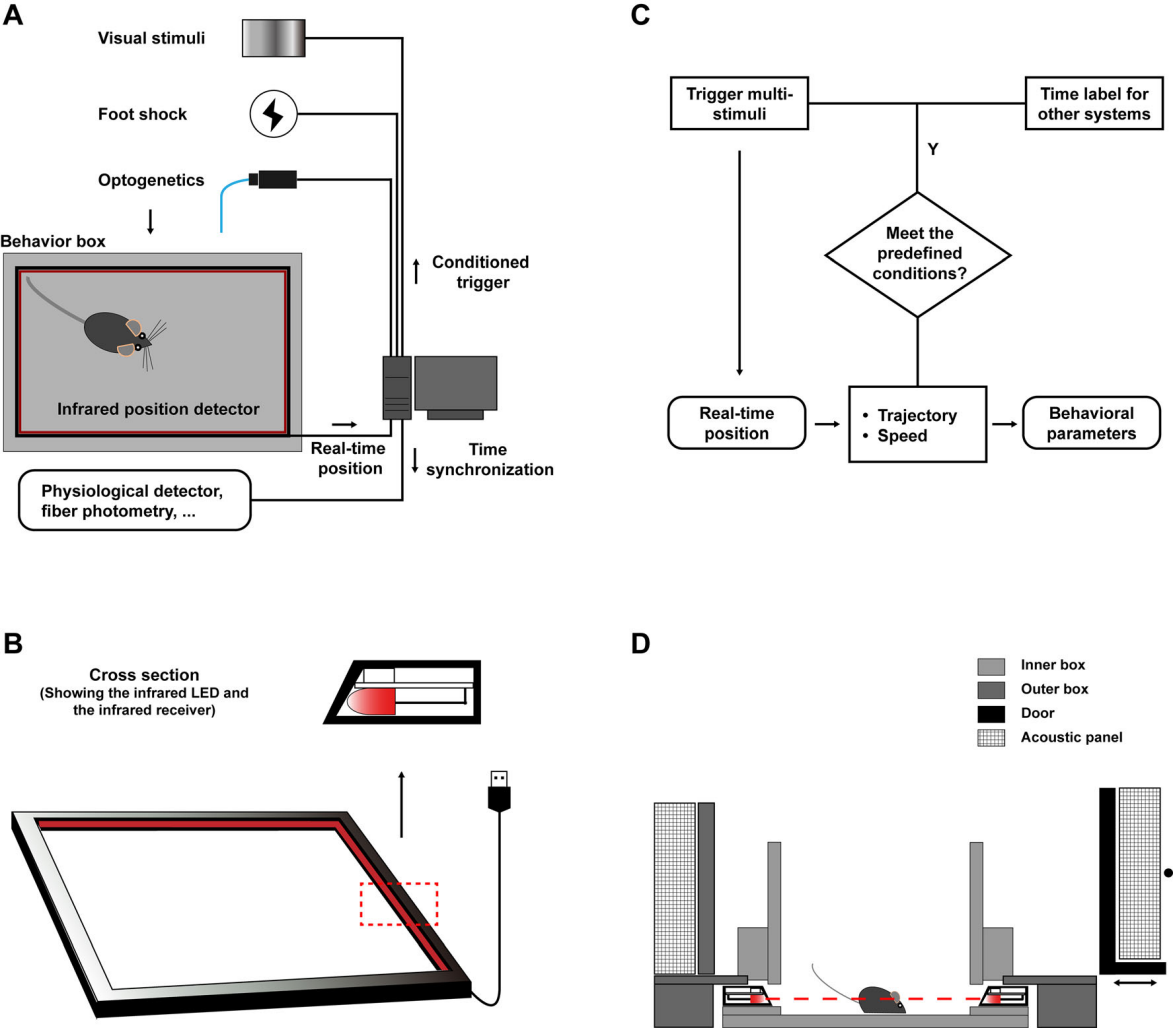
The automatic infrared behavior-monitor (AIBM) system. **A** Schematic of the system. Real-time mouse positions are monitored in the behavior box by an infrared position detector. **B** The infrared real-time position detector is a 42-inch infrared touch frame connected to a computer *via* a USB interface. **C** Flowchart outlining real-time behavioral analysis and closed-loop manipulations. **D** Detailed structure of the behavior box. Several behavioral apparatuses have been adapted and used as the ‘inner box’, all of which are replaceable.

The AIBM system consists of a behavior box, an infrared position detector, and a computer (Fig. 1A). The position detector was a 42-inch frame with infrared detection that is commercially available as a touch screen frame, which detected real-time animal positions. The infrared frame comprised infrared LEDs and infrared receivers and detected animal positions based on shadows cast on the receivers sampled at 60 Hz (Fig. 1B). Positional data were recorded, and two parameters were calculated in real time: trajectory and speed. Based on these parameters, certain predefined conditions were set, and when they were fulfilled, a trigger automatically initiated a series of stimuli or automatically recorded a timestamp on another integrated system (Fig. 1C).

The behavior box provided a stable experimental environment that was refractory to changes in light and noise levels that one may expect to arise due to varying laboratory conditions (Fig. 1D). It had an outer box made of aluminum alloy profiles and acrylic boards, which provided a scaffold. Acoustic panels could be fixed on the outer wall to block room noise and light. Sitting inside this was an inner box that consisted of upper walls, a lower wall-board and the floor. This inner box was replaceable and could be designed to be one of many different behavior apparatuses (Fig. S1). The touch frame was fixed between the upper wall and the lower wall-board so that the infrared detectors were level with the center of the animal being tested. In mouse experiments, this was at a height of 17 mm. There was a 5 mm–7 mm gap between the upper and lower walls of the inner box, between which was the infrared detector frame (Figs 1D, S1). This gap allowed an optimal amount of infrared light to reach the detectors, which were recessed slightly behind the walls. A variety of stimulus generators, including those for foot-shock and optogenetic delivery, are compatible with the AIBM system, as are multiple recording systems, such as fiber-photometry and physiological telemetry (Fig. 1A).

### Spatial Accuracy Evaluation

**Fig. 2:**
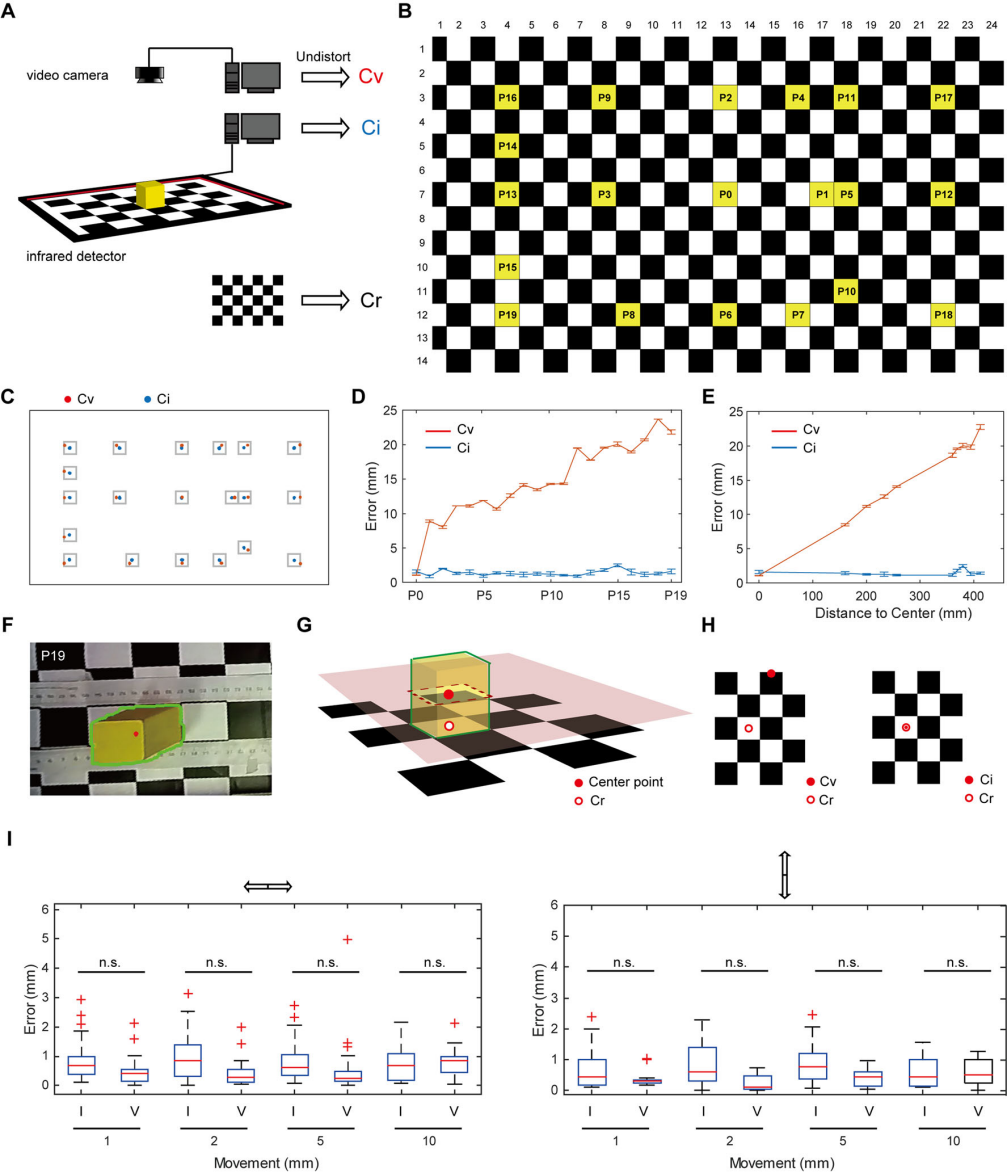
Accuracy of video recording and infrared position detection. **A** Schematic of the experimental set-up. The checkerboard pattern grid is used to set the reference coordinates. The positions of a cube at a series of specific coordinates on the grid are recorded simultaneously by a video camera and the infrared detector. Cr, reference coordinates; Cv, video camera coordinates; Ci, infrared detector coordinates. **B** Yellow grids showing the recorded positions. **C** Positions detected with video camera (red dots) and infrared detector (blue dots). **D** Distance between the recorded coordinates and Cr at each of the 20 positions. *n* = 5 at each point (means and SEM). **E** Distance between the recorded coordinates and Cr across different distances to the center (P0). *n* = 5–20 at each point (means and SEM). **F** Corrected image at P19, showing the shape of the cube detected by video (green frame) and the calculated center point (red dot). **G** Schematic of video recording and infrared detection. **H** Schematic showing the difference between Cv and Ci. **I** Sensitivity of video recording and infrared detector to displacement (I, with infrared detector, V, with video camera; left, cube moved left-to-right, *n* = 12 at each point; right, cube moved top-to-bottom, *n* = 8 at each point; rank sum tests were calculated for comparisons between experimental and control groups. n.s., no significant difference.

To compare the spatial accuracy of the AIBM system with video recording, a checkerboard pattern grid (4 × 4 cm^2^) was used to set the reference coordinates (Cr). A cube (4 × 4 × 4 cm^3^) was moved on the grid, and the positions of the cube were recorded simultaneously by the infrared touch frame and a video camera (Fig. 2A). The center of the cube was taken as its position. The infrared detector coordinates (Ci) and video coordinates (Cv) were compared with the Cr over 20 test positions, P0-P19, numbered according to the distance to the center of view (Fig. 2B). Argus, an open-source software based on the Zhang Zhengyou camera calibration technique [11, 13] was used to undistort video frames. We found that Ci and Cv were similar and both had a small error relative to Cr at the center of view (P0). The Ci error was ~1 mm at all 20 positions, while the Cv error increased with increasing distance to the center (Fig. 2C–E). In the video recording, the 3D cube was recorded as a 2D color-block and Cv was calculated as the center of the shape (Fig. 2F–H). Thus, the deviation caused by the visual angle inevitably increased with distance from the center of view using the monocular video camera. The camera calibration corrected the 2D image, but the shape of the 3D cube in the image was stretched (Fig. 2F), which increased the deviation at the edge of the field-of-view. Compared with these, the infrared detector recorded the cross-section of the cube, and Ci was defined as the center of the cross-section (Fig. 2G, H), which represented the position of the cube with higher accuracy, especially at the border of the recorded area.

To test the spatial resolution of the infrared detector and the video camera, cube displacements of 10 mm, 5 mm, 2 mm, and 1 mm at each position were analyzed (Fig. 2I). The detection error in the infrared recordings was ~1 mm for all displacements at all positions. In the video recordings, the detection error did not significantly differ from that in the infrared recording when the displacement was 1 mm. With increasing displacement, the effect due to deviation of visual angle increased, and there was an increasing trend in detection error. When the displacement was 10 mm, the detection error of the video recordings increased to 1 mm. It bears mentioning that the spatial discrimination of video recording is related to the resolution of the camera. In this experiment, the video resolution was 1920 × 1080, and 1 mm represented with ~2 pixels.

To compare the speed of real-time data manipulation of the infrared detector with video recording, we tracked a remote-controlled car simultaneously with each method. A background subtraction method was used for video tracking [12]. The sampling rate was mainly influenced by computer configuration and video resolution (Table 1). Moreover, to capture clear pictures at a high sampling rate, ambient illumination also needed consideration. The sampling rate of the infrared detector was 60 Hz and was supported by a relatively low-level configuration (Table 1). Ambient illumination did not need to be considered in this case.

**Table 1:** Sampling rate of video recording and the infrared detector in a real-time tracing task.

		Video camera (SY020HD-V1, S-YUE, Shenzhen)	Infrared position detector (42 inches, 10 touch points, Yijing Electronic, Guangdong)
**HEDY IABOX N20, HEDY**	1920 × 1080	12 Hz	60 Hz
Graphics: Integrated	800 × 600	33 Hz	-
CPU: Intel (R) Core (TM) i7-7500U 2.2 GHz			
RAM: 8G			
**Think Centre M910t-D565, Lenovo**	1920 × 1080	24 Hz	60 Hz
Graphics: NVIDA GeForce GT 730	800 × 600	60 Hz	-
CPU: Intel (R) Core (TM) i7-6700 CPU 3.4 GHz			
RAM: 16G			
**Additional settings**		Ambient illumination	-

### Open-loop Paradigms in the AIBM System

**Fig. 3:**
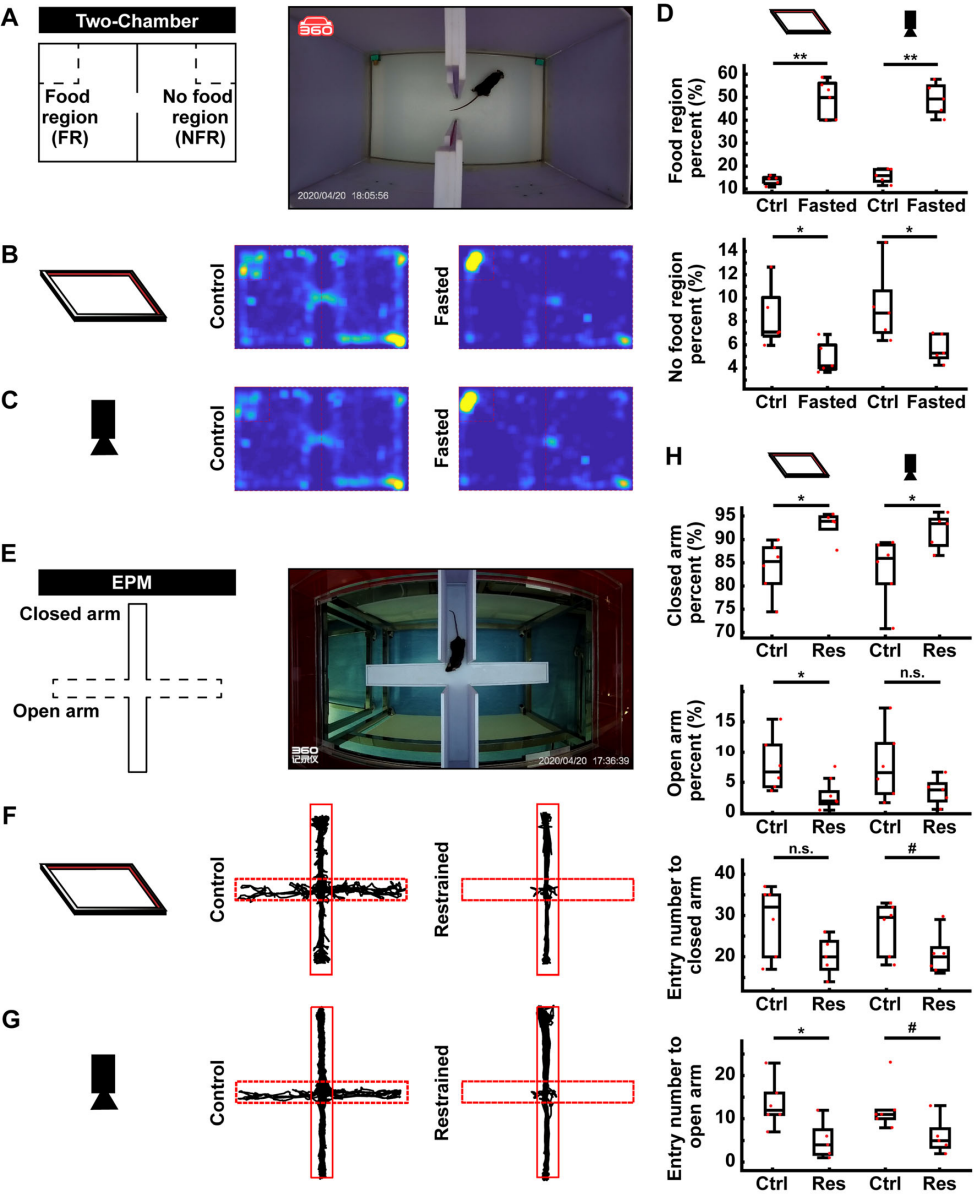
Open-loop paradigms in the AIBM system. **A** Schematic (left) and photograph (right) of the two-chamber paradigm. **B** Representative trajectories of control (left) and the restrained (right) groups collected by the infrared position detector. **C** Representative trajectories of the control (left) and restrained (right) groups collected by video camera. **D** Percentages of time spent in the food (upper) and no-food (lower) region; data collected with the infrared detector (left) and video camera (right). FR, food region; NFR, no-food region. **E** Schematic (left) and photograph (right) of the elevated plus maze (EPM) paradigm. **F** Representative trajectories from control (left) and restrained (right) groups collected by the infrared detector. **G** Representative trajectories from control (left) and restrained (right) groups collected on a video camera. **H** Behavioral parameters in the EPM paradigm. Percentages of time spent in, and the number entries into, the closed/open arm. Data were collected with the infrared position detector (left) and video camera (right). Res, restrained. *n* = 5 mice in each group. #*P* <0.1, *P <0.05, **P <0.01, ***P <0.001, experimental *vs* control groups, rank sum tests.

The AIBM system was tested in mice. Two paradigms that are widely used in neuroscience research, the two-chamber (Fig. 3A) and the EPM (Fig. 3E) paradigms, were used to test the AIBM system. Data recorded by the infrared position detector were compared with those captured by video camera. In the two-chamber paradigm, a holder with food was placed in one corner of the inner box and a holder without food was placed in the opposite corner of the other chamber (Fig. 3A). Mice that had been fasted for one day spent most of their time eating in the food region only, whereas unfasted mice typically explored both chambers (Fig. 3B, C). The percentage of time that the fasted mice spent in the food region was significantly higher than that of the unfasted mice, and the percentage of time spent in no-food regions was significantly lower (Fig. 3D). These data were in line with previous studies [5, 6, 8]. The position distribution of mice recorded with the infrared position detector was similar to that from the video data (Fig. 3B, C).

Another paradigm that is extensively used to generate behavioral indices is the EPM, which is a semi-open environment. Compared to the standard apparatus, the AIBM-EPM (Fig. 3E) became a more closed environment because it was inside the behavior box. We found that the tracking results from the AIBM-EPM showed a similar pattern of results to the video camera data; that is, mice that were restrained prior to the test spent more time in the closed arms and rarely entered the open arms (Fig. 3F–H). These data were consistent with previous results [5, 6]. Thus, multiple open-loop behavioral paradigms are compatible with the AIBM system.

The infrared frame detected the cross section of the body while ANY-maze detected the shape of animal in the analyzed regions. Thus, although the tendency of data from the two methods was the same, the trajectory calculated with the two methods had a small difference.

### Closed-Loop Paradigms in the AIBM System

**Fig. 4:**
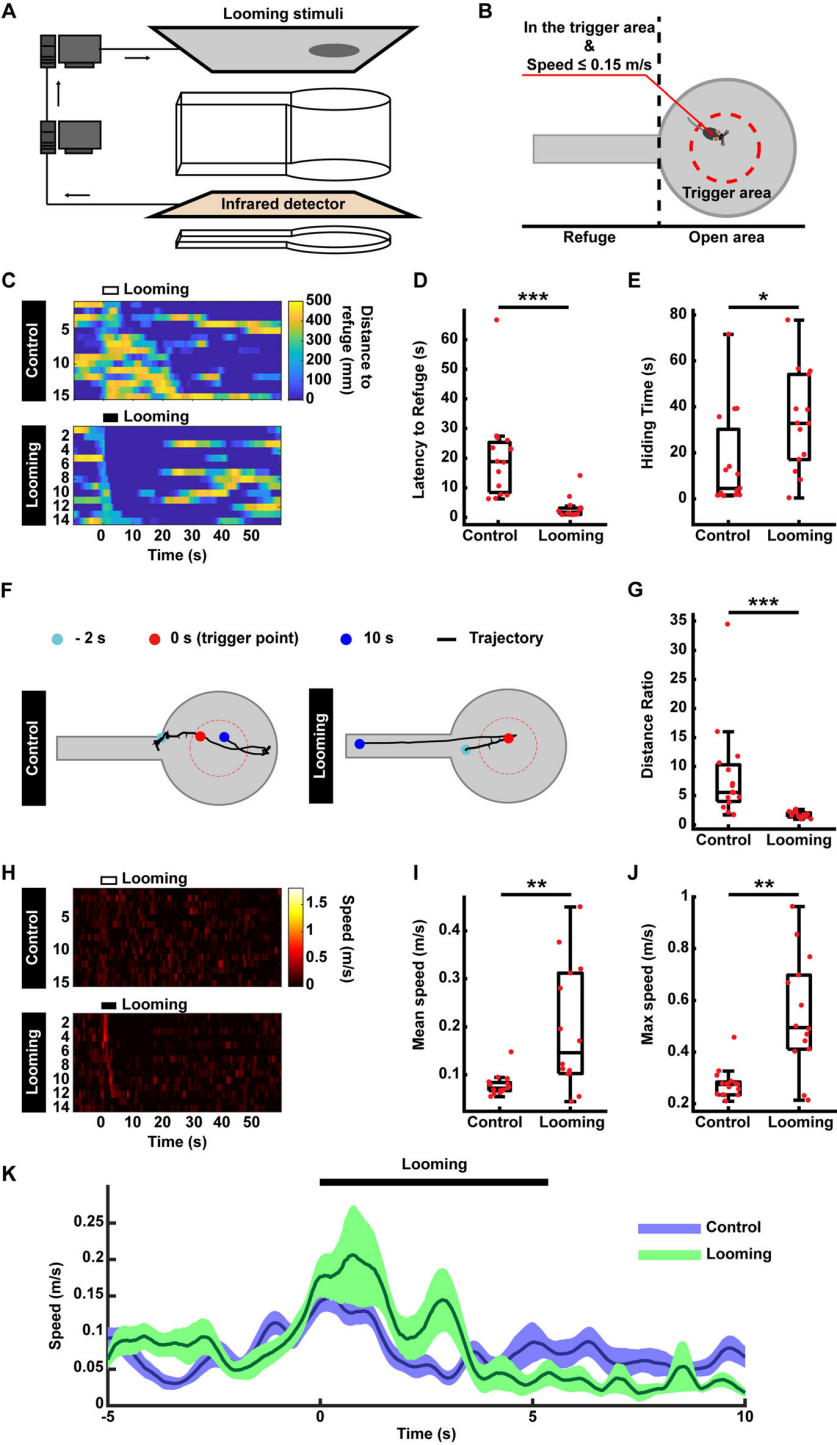
Looming test in the AIBM system. **A** Schematic of the system with the closed-loop looming paradigm. Overhead looming stimuli are triggered *via* serial port communication. **B** The behavior box and trigger conditions for the looming test. **C** The distance of mice to refuge before, during, and after looming stimuli. Upper, negative control group without a looming stimulus. Lower, the looming stimulus group. The duration of the looming stimulus was 5.5 s. **D** Latency to refuge following looming stimuli. **E** Time spent hiding in the refuge after flight. **F** Representative trajectory from the negative control group (left) and the looming group (right). **G** The distance ratio of the trajectory to the refuge, defined as the length of the flight trajectory/straight-line distance from the trigger point to the center of refuge entry. **H** Speed of mice to the refuge before, during, and after looming stimuli. Upper, negative control group without looming stimuli. Lower, looming group. The duration of the looming stimulus was 5.5 s. **I** Mean speed to refuge following looming stimuli. **J** Maximum speed to refuge following looming stimuli. **K** Average speed curve of mice before, during, and after looming. *n* = 15 mice in the negative control group, *n* = 14 in the looming group. *P <0.05, **P <0.01, ***P <0.001, experimental *vs* control groups, rank sum tests.

To test the automatic trigger feature of the AIBM system, the looming test paradigm was performed. In this paradigm, looming stimuli were presented to induce flight behavior in mice [14–17]. Looming stimuli were presented on an LCD monitor above the behavior box (Fig. 4A). The inner box was designed with a circular, open area connected to a narrow refuge. The center of the open area was a trigger area. After 5 min of habituation, a looming stimulus was set to trigger when a mouse’s behavior matched predefined conditions, namely, entering the trigger area at a speed no more than 0.15 m/s (Fig. 4B). The trigger signal was delivered *via* serial port communication. In a negative control group, no looming stimulus was presented.

Distance to the refuge (Fig. 4C), latency to the refuge (Fig. 4D), and time spent hiding (Fig. 4E) were calculated using real-time mouse positions. Consistent with previous results [14–18], looming stimuli elicited a fast escape to the refuge (Fig. 4C, D) where a mouse would stay hidden for many seconds (Fig. 4E). Thus, the AIBM system worked extremely well for closed-loop behavioral tests. Stimuli were automatically triggered, thereby increasing the overall efficiency of the experimental process. Moreover, putative bias and human experimenter error were excluded.

Mouse trajectories were recorded in real time with the infrared position detector. Without the deviation due to the visual angle in the video data, the trajectories were comparatively accurate, and thus well-suited for the analysis used to evaluate flight behavior (Fig. 4F, G). In the negative control group, mice explored the inner box, whereas in the looming group, mice ran directly to the refuge in response to the looming stimulus (Fig. 4F). We used a distance ratio to compare flight responses, defined as the length of the flight trajectory divided by the most direct, straight-line distance from the trigger point to the center of the refuge entry. The distance ratio for the looming group was significantly lower than that of the negative control group (Fig. 4G), indicating the mice ran directly to the refuge in response to looming. Furthermore, speed was calculated in real time based on accurate real-time mouse positions (Fig. 4H). Mean and maximum speeds were analyzed (Fig. 4I, J) in addition to average speed curves across trials (Fig. 4K). Thus, accurate trajectory and speed can be recorded using the AIBM system.

In addition to visual stimuli, other sensory stimuli can be integrated into the AIBM system, including foot-shock. We replaced the acrylic floor with a foot-shock board to test this. The same trigger conditions as for the looming test were used to start the foot-shock automatically. In the foot-shock group, the current was set 0.5 mA. In a negative control group, no foot-shock was delivered. Foot-shocks were set to stop automatically when a mouse entered the refuge. Such start and stop signals were delivered *via* TTL signals using a DAQ card (Fig. S2A). The same parameters as those in the looming paradigm were used to represent mice behavior.

Foot-shock elicited escape to refuge, which took no more than 2 s (Fig. S2B, C) at high speed (Fig. S2G–J), followed by hiding in the refuge for some minutes (Fig. S2B, D). The distance ratio of the foot-shock group was significantly lower than that of the negative control group (Fig. S2E). However, in some cases in this control group, although the latency to the refuge was short, trajectories showed that, rather than make a U-turn to the refuge as soon as the foot-shock was triggered, mice continued moving forward, ran to the wall, and then followed it round to the refuge (Fig. S2F). This shows that the AIBM system is able to accurately detect and record behavior triggered by different sensory stimuli.

### The Optogenetic-AIBM System

**Fig. 5:**
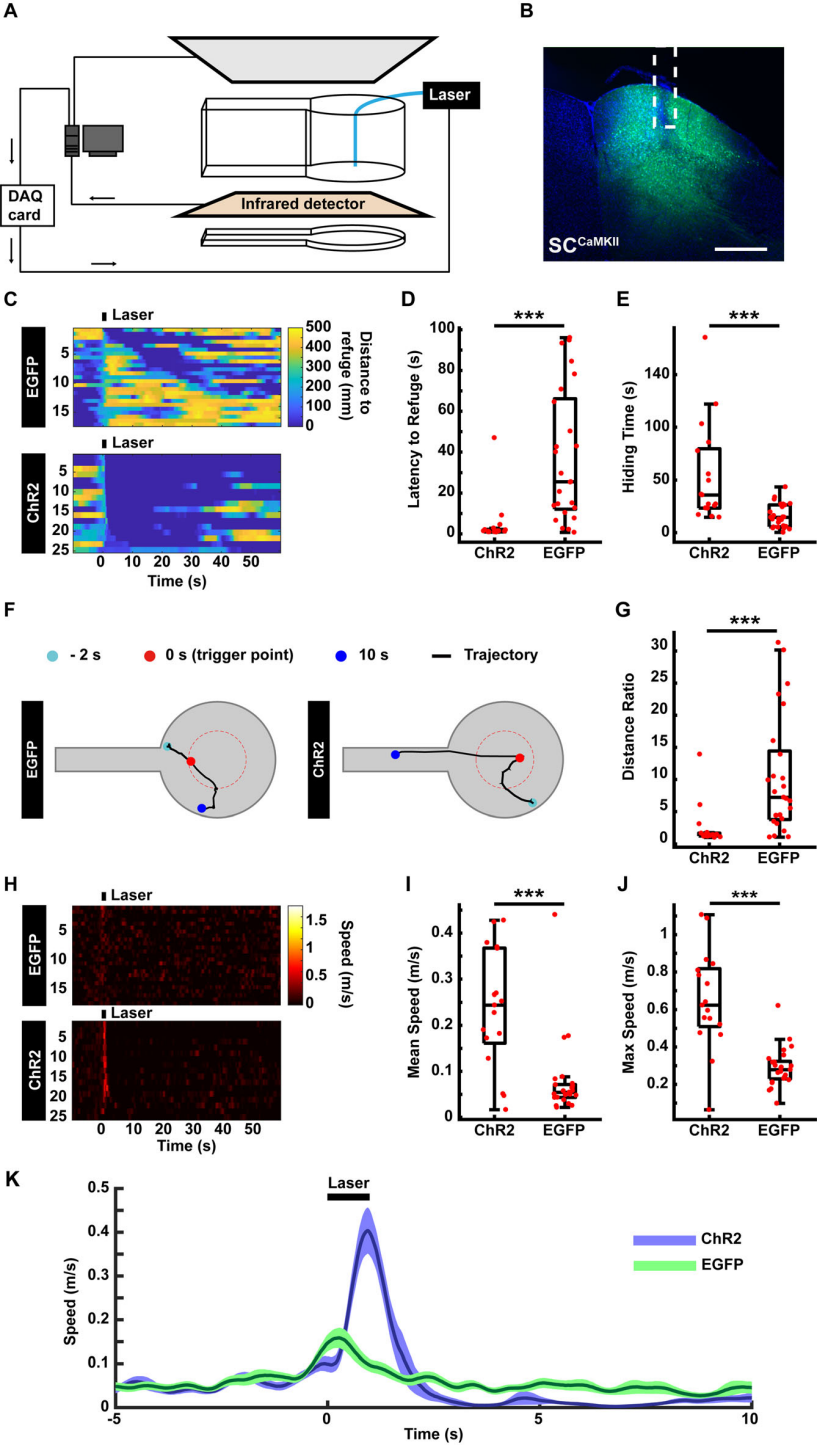
Optogenetic test in the AIBM system. **A** Schematic of the optogenetic-AIBM system. The optogenetics laser was triggered by the data acquisition (DAQ) card. **B** Expression of the AAV-CaMKII-ChR2-mcherry virus in the SC and the optic fiber position (white dotted line). Scale bar, 500 µm. **C** Distance of mice to refuge before, during, and after optogenetic stimuli. Upper, control group expressing AAV-CaMKII-EGFP in the SC. Lower, ChR2 group expressing AAV-CaMKII-ChR2-EGFP in the SC. Laser stimuli lasted 1 s. **D** Latency to the refuge following optogenetic stimuli. **E** Time spent hiding in the refuge after optogenetic stimuli. **F** Representative trajectories of the negative control group (left) and the optogenetic group (right). **G** Distance ratio of the returning trajectory. **H** Speed of mice to the refuge before, during, and after optogenetic stimuli. Upper, the negative control group. Lower, the ChR2 group. **I** Mean speed when returning to the refuge following optogenetic stimuli. **J** Maximum speed when returning to the refuge following optogenetic stimuli. **K** Average speed curve before, during, and after optogenetic stimuli. *n* = 25 trials from 5 mice in the control group. *n* = 17 trials from 6 mice in the ChR2 group. ***P <0.001, experimental *vs* control groups, rank sum tests.

Optogenetic tools activate or inhibit specific neurons on a millisecond time scale, and thus are widely used to clarify the functions of specific neural circuits in regulating a given behavior in real time [19, 20]. Here, we integrated an optogenetics rig into the AIBM system where the optic fiber entered the behavior box from above. Laser stimuli were triggered automatically according to predefined conditions. The automatic trigger signal was translated into a TTL signal through a DAQ card and delivered to the optogenetic control system to trigger laser stimuli (Fig. 5A). We used mice in which the superior colliculus (SC) was infected with AAV-CaMKII-ChR2-EGFP or AAV-CaMKII-EGFP virus and stimulated by a blue laser (473 nm, Fig. 5B). Latency to the refuge (Fig. 5C, D), time spent hiding (Fig. 5C, E), mouse trajectories (Fig. 5F, G) and speed (Fig. 5H–K) were analyzed. In line with a previous report [10], activation of the SC induced strong flight behavior. Thus, the optogenetic-AIBM system is able to automatically manipulate neural activation using optogenetics and detect behavioral effects.

### The AIBM System Combined with Other Experimental Systems.

**Fig. 6:**
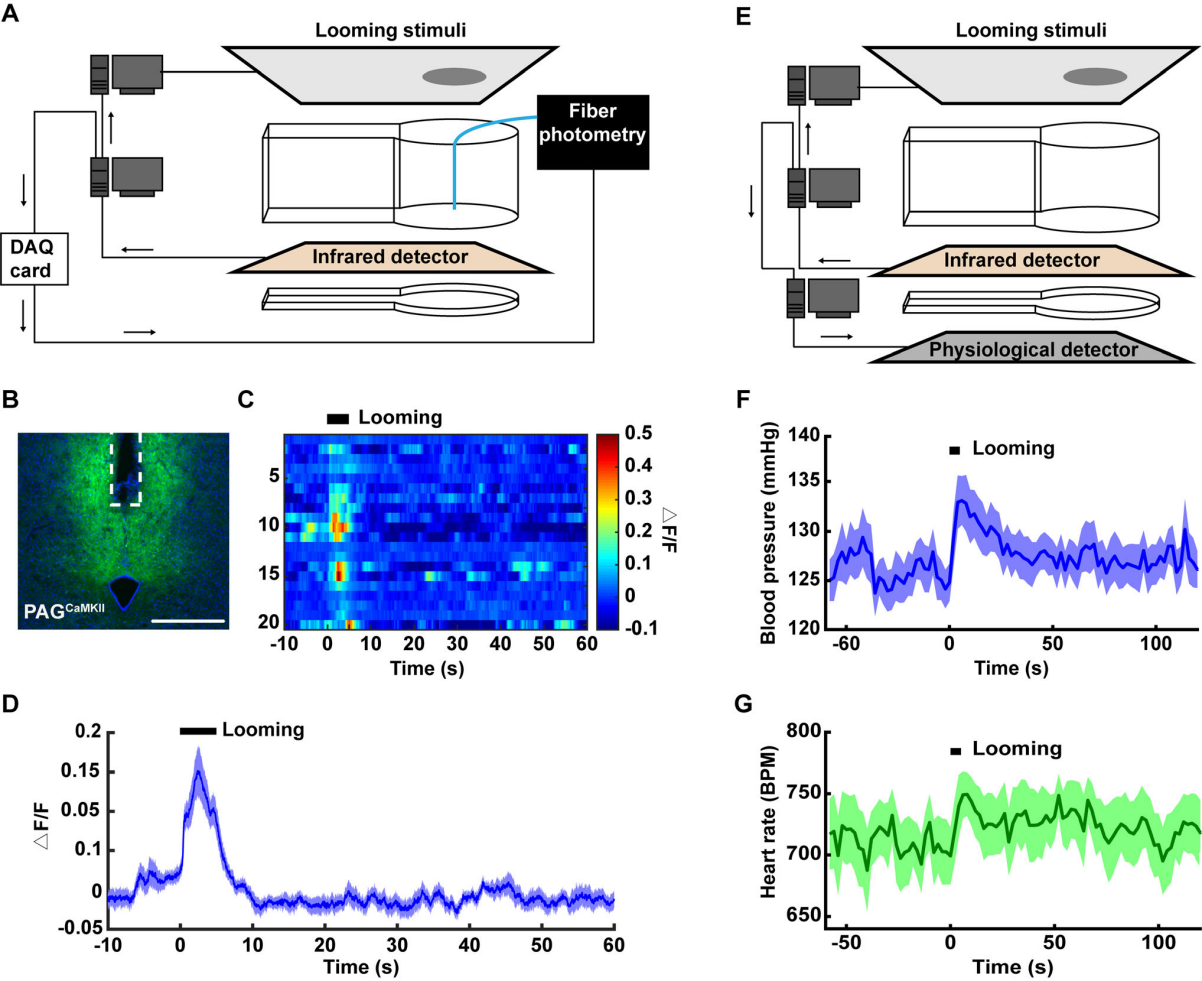
The AIBM system integrated with the looming stimuli test combined with fiber photometry recording or blood pressure measurement. **A** Schematic of the AIBM system with the looming test paradigm combined with a fiber-photometry recording system. Looming stimuli were triggered through the serial port and time synchronization between the AIBM system and fiber photometry were through the data acquisition (DAQ) card. **B** Expression of AAV-CaMKII-GCaMP6 virus in the dPAG and optic fiber position (white dotted line). Scale bar, 500 µm. **C** Ca^2+^ responses during each trial before, during, and after looming stimuli. The duration of the looming stimulus was 5.5 s. Each line represents one trial of the looming test. *n* = 20 trials from 4 mice. **D** Ca^2+^ response curve. Line, mean Ca^2+^ response; shadow, SEM. **E** Schematic of the AIBM system with the looming test paradigm combined with a blood pressure measurement system (DSI, MN). Looming stimuli were triggered through a serial port, as was the time synchronization between the AIBM system and the blood pressure measurement system. **F** Blood pressure curve. Line, mean blood pressure; shadow, SEM. **G** Heart rate curve. Line, mean heart rate; shadow, SEM. *n* = 14 trials from 4 mice. Each dot represents the result of 1 looming test trial. *P <0.05, **P <0.01, ***P <0.001, experimental *vs* control groups, rank sum tests.

Multiple signals, such as electrical or Ca^2+^ responses in the brain, or other physiological changes in the body, are often recorded simultaneously with behavioral recordings. To meet these requirements, the AIBM system was designed to easily integrate with other experimental systems. First, a fiber photometry system was used along with the looming stimulus test to record looming-induced Ca^2+^ responses in mouse brains. An automatic trigger signal was used to label events. The trigger signal was translated into a TTL signal by a DAQ card, delivered to the fiber photometry system, and then recorded using software (Fig. 6A). It had been reported that dorsal periaqueductal grey (dPAG) neurons respond to looming stimuli [15], and to test the system we therefore recorded Ca^2+^ signals in dPAG^CaMKII^ neurons before, during, and after looming (Fig. 6B). Notable Ca^2+^ responses induced by looming were found (Fig. 6C, D). Furthermore, a blood pressure system (DSI, MN), which detected blood pressure and heart rate in real time, was combined with the AIBM system. For this, a pressure transmitter (PA-C10, DSI, MN) was implanted into the carotid artery in mice. Pressure signals were transmitted wirelessly *via* radio-frequency signals (Fig. 6E). Blood pressure and heart rate increased in response to the looming stimulus (Fig. 6F, G). These data provided physiological indices that can be used to evaluate fear induced by looming, suggesting possibilities for the ABIM system to be combined with various physiological measurement systems.

## Discussion

Here, we describe an automatic infrared behavior-monitor system, which detects the real-time position of freely-moving animals with an infrared touchscreen frame, can be configured to automatically trigger external stimuli according to strict predefined conditions, and is compatible with multiple paradigms within a stable experimental environment.

Capacitance touch screens, such as the iPad^TM^ or Android^TM^ tablets have been used in animal behavior studies [21]. In addition to trajectory recording, tablets can track animal paws for gait analysis, and can thus be immensely helpful in subtle behavioral analysis. However, since in these cases the tablet was used as the floor of the behavioral apparatus, foot-shock related paradigms (e.g. fear conditioning), elevated paradigms (e.g. the EPM and elevated zero-maze), or paradigms involving holes in the floor (e.g. the Barnes maze), which are all compatible with the AIBM system, are not suitable for capacitance touchscreen systems. Moreover, the high price of large tablet PCs may also limit their application.

Video recording, the most common method in behavioral data acquisition, records the entire experimental process. Large amounts of information can be extracted from videos, such as animal trajectories, posture, the facial expressions, and even whisker movements [2, 22–28]. Based on the rapid development of computer vision and machine learning, numerous algorithms have been developed to extract more and more information from videos, as well as increasing processing speed. For animal tracking, the AIBM system provides a highly efficient and low-cost solution as an alternative to video-based tracking. First, to reduce distortion of trajectories, on the one hand, the video camera should be directly above the behavioral chamber with the main ‘action area’ at the center of the frame. Some paradigms have no physical space for video recording from above [17, 29], in which case, the only other option is a side-view recoding. In these cases, the deviation due to visual angle (Fig. 2C–H) reduces the accuracy of trajectory recording, and further affects calculations of speed. On the other hand, multiple or binocular cameras can be used to shoot from different visual angles, and advanced algorithms can be applied to modeling and locating the animal [11, 25, 26, 30], which would increase the difficulty of both data acquisition and analysis. In the AIBM system, the position detection has no distortion issue, and the comparatively accurate trajectory and speed can be recorded in a relatively simple manner. Second, video data manipulation requires computational power. Manipulating video data in real time requires highly-configured, hardwired, and professional algorithms, which places limitations on image quality and processing speed (Table 1).

There are also limitations in the AIBM system. First, the AIBM system was not designed to detect subtle behaviors. The infrared touchscreen frame records a cross-section of the animal. The head, tail, or paws cannot be tracked individually. Only limited behaviors such as flight, freezing, and rearing can be identified based on changes in the patterns of the cross-sectional area and position [31]. In addition, the AIBM system is not suitable for multiple-animal paradigms, such as aggression tests [32, 33]. This is because it is not possible to resolve the trajectories of multiple animals in close proximity since the information recorded by the infrared detector is insufficient to distinguish between animals. The compatibility of some frequently used behavioral tests with the AIBM system is listed in Table 2.

**Table 2:** Compatibility of some behavioral tests with the AIBM system.

Behavioral tests				Compatibility	Notes
Free moving	Single animal	Intact floor	Open field [6, 7]	√	
			Two-chamber [8–10]	√	
			Three-chamber (novel location/object recognition ) [4, 34, 35]	√	Theoretically possible
			Light-dark box [4, 36, 37]	√	Theoretically possible
			Y maze [38, 39]	√	Theoretically possible
			T maze [40, 41]	√	Theoretically possible
			Radial maze [42, 43]	√	Theoretically possible
		Elevated	Elevated plus maze [4–7]	√	
			Elevated zero maze [43]	√	Theoretically possible
		Foot-shock pad as the floor	Fear conditioning [44, 45]	√	
		Holes on the floor	Barnes maze [46, 47]	√	Theoretically possible
			Cheeseboard maze [4, 39, 48]	√	Theoretically possible
		Test in water	Morris water maze [49, 50]	×	Water may influence the localization accuracy
	More than one animal	One animal free moving	Social recognition [4, 51, 52]	√	Theoretically possible
			Predator fear [53, 54]	√	Theoretically possible
		All animals free moving	Resident-intruder aggression [32, 33]	×	Insufficient to distinguish between animals
Fixed				×	No trajectory

√, compatible, ×, incompatible.

In summary, the AIBM system represents a simple and accurate strategy in trajectory tracking, to achieve the high-throughput data acquisition and high efficiency of data manipulation required in modern behavioral settings at low cost.

## Supplementary Information

Below is the link to the electronic supplementary material.

Supplementary file1 (PDF 546 KB)
