# Supplementary material for: An Infrared Touch System for Automatic Behavior Monitoring
Source: Neurosci Bull. 2021 Mar 31;37(6):815–30. doi: 10.1007/s12264-021-00661-4 (PMC8192659; doi:10.1007/s12264-021-00661-4)
Supplement: Supplementary file 1 — Supplementary file1 (PDF 546 KB) [file 12264_2021_661_MOESM1_ESM.pdf]

Supplementary Materials

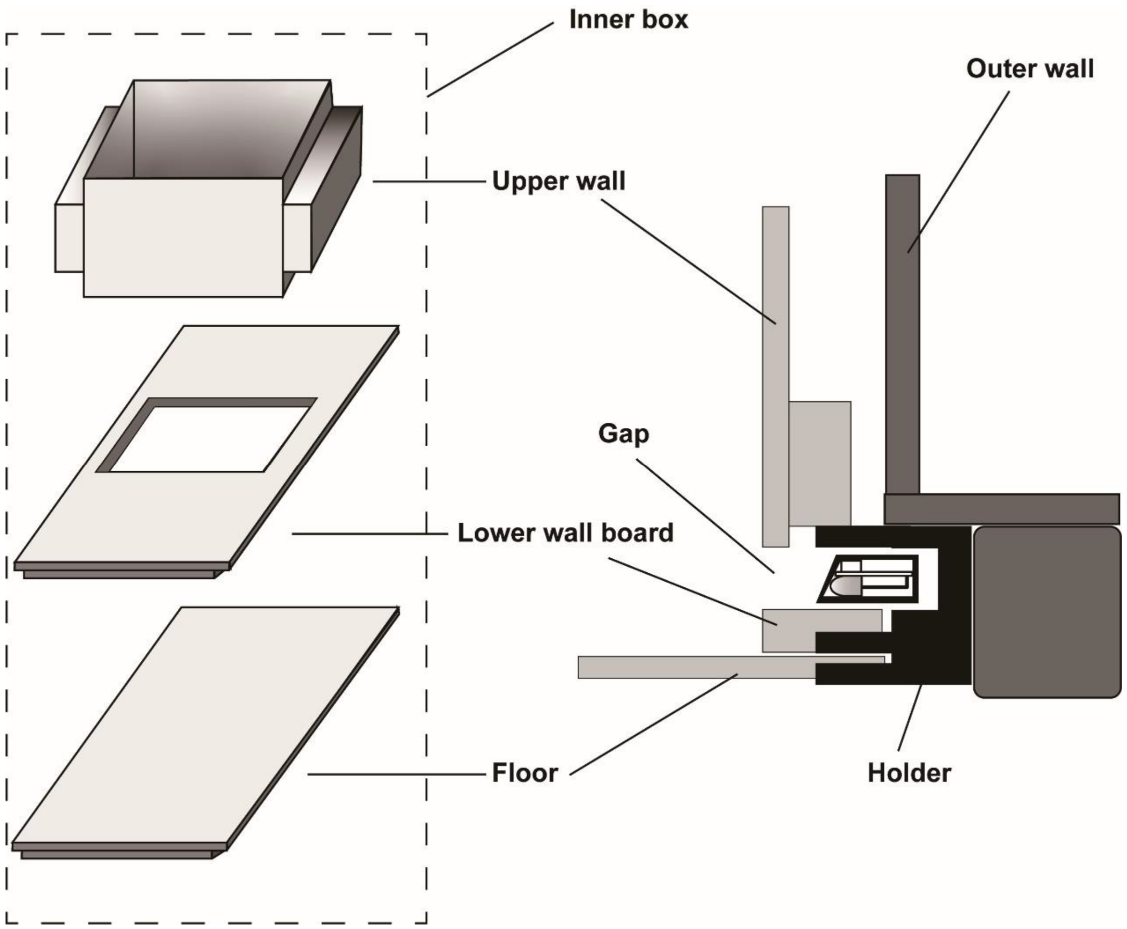

Fig. S1 The inner box and the behavior box holder. The open field test apparatus as an example of an 'inner box'. Schematic showing how the upper wall, lower wall board, and floor are combined and fitted into the box holder.

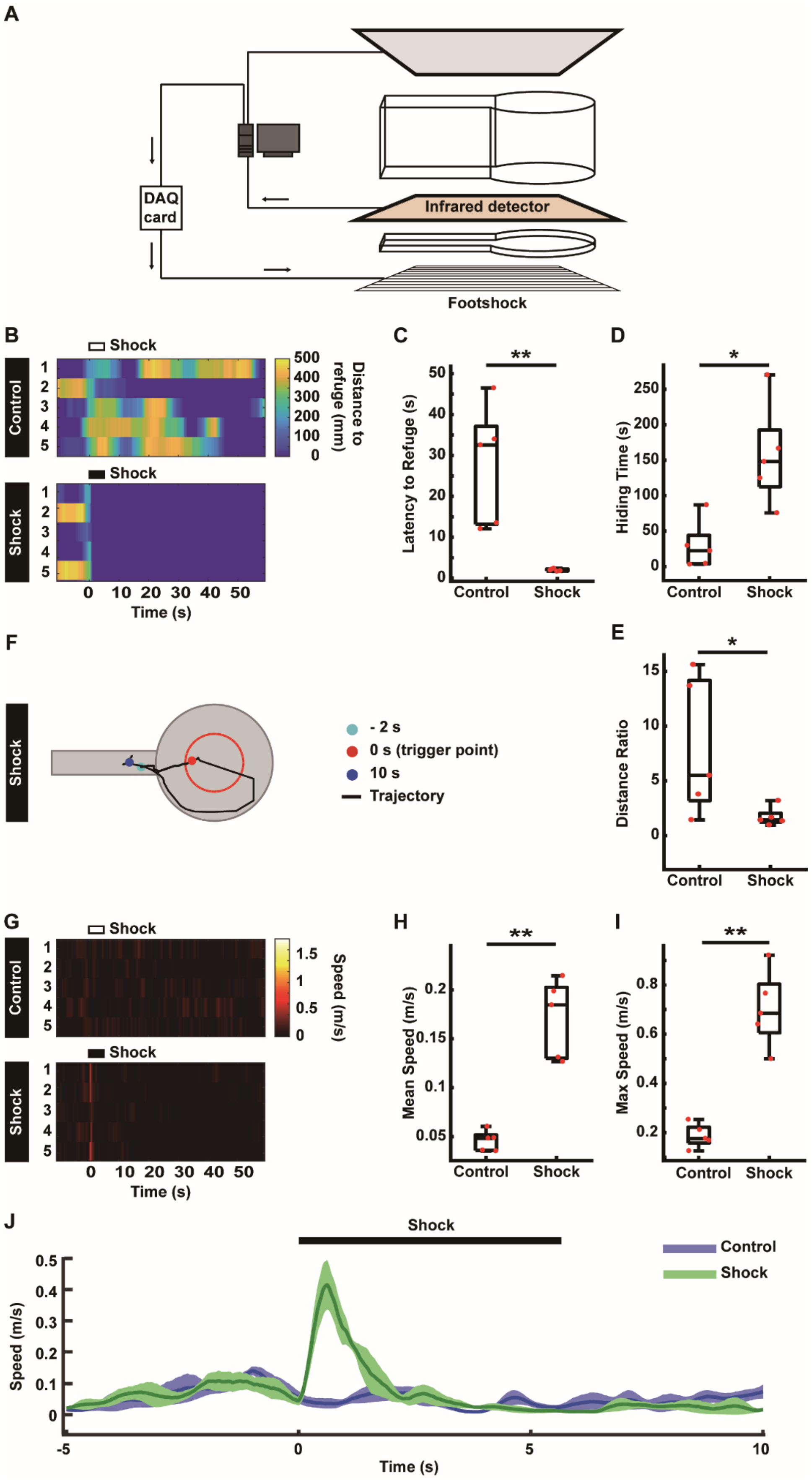
A
DAQ card
Infrared detector
Footshock
B
Shock
Control
Shock
Distance to refuge (mm)
Time (s)
C
Latency to Refuge (s)
Control
Shock
D
Hiding Time (s)
E
Distance Ratio
F
- 2 s
0 s (trigger point)
10 s
Trajectory
G
Speed (m/s)
H
Mean Speed (m/s)
I
Max Speed (m/s)
J
Speed (m/s)
Time (s)

Fig. S2 Related to Fig. 3. Foot-shock test in the AIBM system. **A** Schematic of the AIBM system with the closed-loop foot-shock paradigm. The foot-shock is triggered through the data acquisition (DAQ) card. **B** Distance of mice to refuge before, during, and after foot-shock. Upper, negative control group (no foot-shocks); lower, foot-shock group (0.5 A for 6 s). **C** Latency to refuge following foot-shock. **D** Time spent hiding in the refuge following foot-shock. **E** Distance ratio of the trajectory to refuge. **F** An example trajectory showing a mouse that, after triggering the foot shock, ran forward first and entered the refuge *via* a long circuitous path. **G** Speed of mice to the refuge before, during, and after foot-shock. Upper, negative control group (no foot-shocks); lower, foot-shock group (0.5 A for 6 s). **H** Mean speed to refuge following foot-shock. **I** Maximum speed to refuge following foot-shock. **J** Average speed curve before, during, and after foot shock. n = 5 mice in each group. *P <0.05, **P <0.01, ***P <0.001 *vs* negative control group.
